# Urinary Calculi and Risk of Cancer: A Nationwide Population-Based Study

**DOI:** 10.1097/MD.0000000000000342

**Published:** 2014-12-02

**Authors:** Chia-Jen Shih, Yung-Tai Chen, Shuo-Ming Ou, Wu-Chang Yang, Tzeng-Ji Chen, Der-Cherng Tarng

**Affiliations:** From the Department of Medicine (C-JS), Taipei Veterans General Hospital, Yuanshan Branch, Yilan; School of Medicine (C-JS, Y-TC, S-MO, W-CY, T-JC, D-CT), National Yang-Ming University; Department of Medicine (Y-TC), Taipei City Hospital Heping Fuyou Branch; Division of Nephrology (S-MO, W-CY, D-CT), Department of Medicine; and Department of Family Medicine (T-JC), Taipei Veterans General Hospital, Taipei, Taiwan.

## Abstract

Previous studies have shown that urinary calculi are associated with increased risks of urinary tract cancers. However, the association between urinary calculi and overall cancers is a largely undefined body of knowledge.

We conducted a nationwide population-based cohort study using Taiwan's National Health Insurance Research Database from 2000 and 2009. Patients were excluded if they had antecedent cancers or urinary calculi before the enrollment. All study subjects were followed until the occurrence of cancer, dropout from the NHI program, death, or the end of 2010. Patterns of cancer incidence in patients with urinary calculi were compared with those of the general population using standardized incidence ratio (SIR).

A total of 43,516 patients with urinary calculi were included. After a median follow-up of 5.3 years, 1891 patients developed cancer. The risk of overall cancers was significantly increased (SIR, 1.75; 95% confidence interval [CI], 1.68–1.83). We observed that urinary calculi was associated with higher risk of cancers of kidney (4.24; 95% CI, 3.47–5.13), bladder (3.30; 95% CI, 2.69–4.00), thyroid (2.50; 95% CI, 1.78–3.40), hematologic origin (2.41; 95% CI, 1.92–2.99), breast (1.84; 95% CI, 1.54–2.20), lung (1.82; 95% CI, 1.59–2.07), digestive tract (1.69; 95% CI, 1.57–1.82), and head and neck (1.54; 95% CI, 1.32–1.79), respectively.

Our study shows that urinary calculi are associated with higher risk of systemic cancers in addition to urinary tract cancers. Further study is required to validate this association.

## Abstract 2

Supplemental Digital Content is available in the text

## INTRODUCTION

Urinary calculus is a common disorder with lifetime incidence of 8.8% to 12% in general population.^1,2^ The cost estimate for intervention of urinary calculi could be >2 billion US dollars in a year.^3^ In United States, the prevalence of urinary calculi has increased from 3.8% in 1976 to 1980^4^ to 8.8% in 2007 to 2010.^2^ Moreover, due to dietary westernization, the effect of urinary calculi on public health issue is overwhelming around the world.^5^

Ample evidence of animal studies has shown that urinary tract calculi lead to urinary tract cancer formation.^6,7^ Supersaturation of concentrated carcinogens with urinary calculi may play a crucial role in tumorigenesis. Moreover, chronic obstruction, inflammation or infection induced by urinary calculi may possibly contribute to tumor growth. Long-term exposure of inflammation and accumulation of carcinogens, especially in asymptomatic urinary calculi, might lead to subsequent cancer development. The increased risks of urinary tract cancers associated with urinary calculi have been reported in previous population-based studies.^8,9^ However, the association of urinary calculi with systemic cancers in humans is a largely undefined body of knowledge.

Recent published studies disclosed that urinary calculi were associated with other systemic disorders such as coronary heart disease, diabetes mellitus, and even metabolic syndrome.^10–13^ Accordingly, urinary calculus became known as a systemic disorder rather than a local disease. As chronic inflammation has been known to be associated with increased risk of cancer development,^14–16^ it prompted us to investigate whether patients with urinary calculi are associated with a higher risk of systemic cancer development in a nationwide population-based study using Taiwan's National Health Insurance Research Database (NHIRD).

## MATERIALS AND METHODS

### Data Source

In this nationwide cohort study, we used the Longitudinal Health Insurance Database (LHID) from 1995 to 2010 obtained from the NHIRD. National Health insurance (NHI) program was launched in Taiwan in 1995, which contains health care data from >99% of the population of 23 million people. The LHID consisted of 1 million beneficiaries randomly sampled from the original NHI beneficiaries. The LHID consists of deidentified secondary data released for research purposes. The database comprises comprehensive information, including the entire registry and claims data from this health insurance system, ranging from demographic data to detailed orders from ambulatory and inpatient care. The accuracy of diagnoses in the NHIRD has been validated for several diseases.^17–20^ Several published papers have used the NHIRD as the basis for their studies.^21–23^ The diseases were coded according to the International Classification of Disease, Ninth Revision, Clinical Modification (ICD-9-CM) diagnosis codes.

This study followed the Declaration of Helsinki on medical protocol. Due to the retrospective nature of this study with deidentified secondary data, it was exempt from full review by the Institutional Review Board of Taipei City Hospital (TCHIRB-1030409-W).

### Study Subjects

From January 1, 2000 to December 31, 2009, patients with a diagnosis of urinary calculi were enrolled. The diagnosis of urinary calculi was defined as patients with at least twice ambulatory visit or hospitalization coding ICD-9-CM 592.x or 594.x. We included incident urinary calculi patients who were >18 years old. We excluded patients with antecedent cancers and a diagnosis of any cancer within the first year of the follow-up period because of surveillance bias. The index date was defined by 366 days after first-time diagnosis of urinary calculi in order to avoid immortal time bias. Information regarding comorbidities including Charlson Comorbidity Index (CCI) score, diabetes mellitus, hypertension, chronic kidney disease, coronary artery disease, dyslipidemia, and chronic liver disease was collected for analysis. To define the disease severity, we collected data of stone location and treatment procedure for stone within 3 months after diagnosis of urinary calculi. Data regarding monthly income levels were collected as a surrogate of economic status and times of ambulatory visit in the past 1 year were also collected as a marker of health care utilization. The urbanization levels of the residential area were also gathered.

### Outcomes

The endpoint of the current study was any cancer occurrence. To identify a patient diagnosed with cancer, we used the data from the Taiwan's catastrophic illness registry to which pathohistologic confirmation for a diagnosis of cancer is required to be reported. Patients in Taiwan's catastrophic illness registry can be exempted from related medical expenses and therefore the barrier for cancer registration is minimized. All patients were followed until the occurrence of cancer, dropout from the NHI program, death, or the end of 2010.

### Statistical Analysis

The risk of cancer among the patients with urinary calculi was determined with the standardized incidence ratio (SIR), which is defined as the ratio of the observed to the expected cancer numbers. The expected numbers of cancers was calculated by adding up the national incidence rate of cancers according to age (in 5-year intervals), sex, and calendar year by the corresponding stratum-specific person-time accrued in the cohort. The population of each age and sex strata and the corresponding stratum-specific incidence rates of cancer for the entire Taiwanese population were based on the population census and cancer registry data from 2000 to 2010, respectively. The 95% confidence intervals (CIs) for the SIRs were estimated under the assumption that the observed number of cancers followed a Poisson probability distribution. Besides, stratified analyses of SIRs for the subgroups according to sex, age, duration after enrollment, urinary stone location, and treatment procedures for stone were performed.

Univariate and multivariate Cox regression models using backward elimination were used to analyze the association between the characteristics of urinary calculi and cancer, and to identify predictors of cancer development among patients with urinary calculi. Risk factors with a *P* value <0.1 were entered into the multivariate analysis. Microsoft SQL Server 2008 R2 (Microsoft Corp, Redmond, WA) was used for data linkage, processing, and sampling. All statistical analyses were conducted using STATA statistical software (version 12.0; StataCorp, College Station, TX). A *P* value <0.05 was considered to be statistically significant.

### Sensitivity Analysis

To assess the reliability of our findings, we further conducted the following analyses. First, we conducted analyses for those with their index date in 2000 to 2004 and 2005 to 2009 separately to look for any evidence of a cohort effect. Second, we conducted a series of analyses applying different criteria to enroll patients who had been newly diagnosed cancer within 90 days or 180 days to minimize misclassification bias. These sensitivity analyses were applied to evaluate consistency of the association between urinary calculi and the risk of cancer.

## RESULTS

### Characteristics of the Study Population

During a 10-year period, 43,516 patients with a diagnosis of urinary calculi were identified and met the inclusion criteria. The mean follow-up was 5.3 ± 2.9 years, and the entire cohort was observed for 229,238 person-years from 2000 to 2010. Mean age of all patients at the time of diagnosis of urinary calculi was 48.8 ± 14.7 years. Of these patients, male patients (65.4%) were predominant. Other demographic characteristics and clinical aspects are shown in Table 1.

**TABLE 1:**
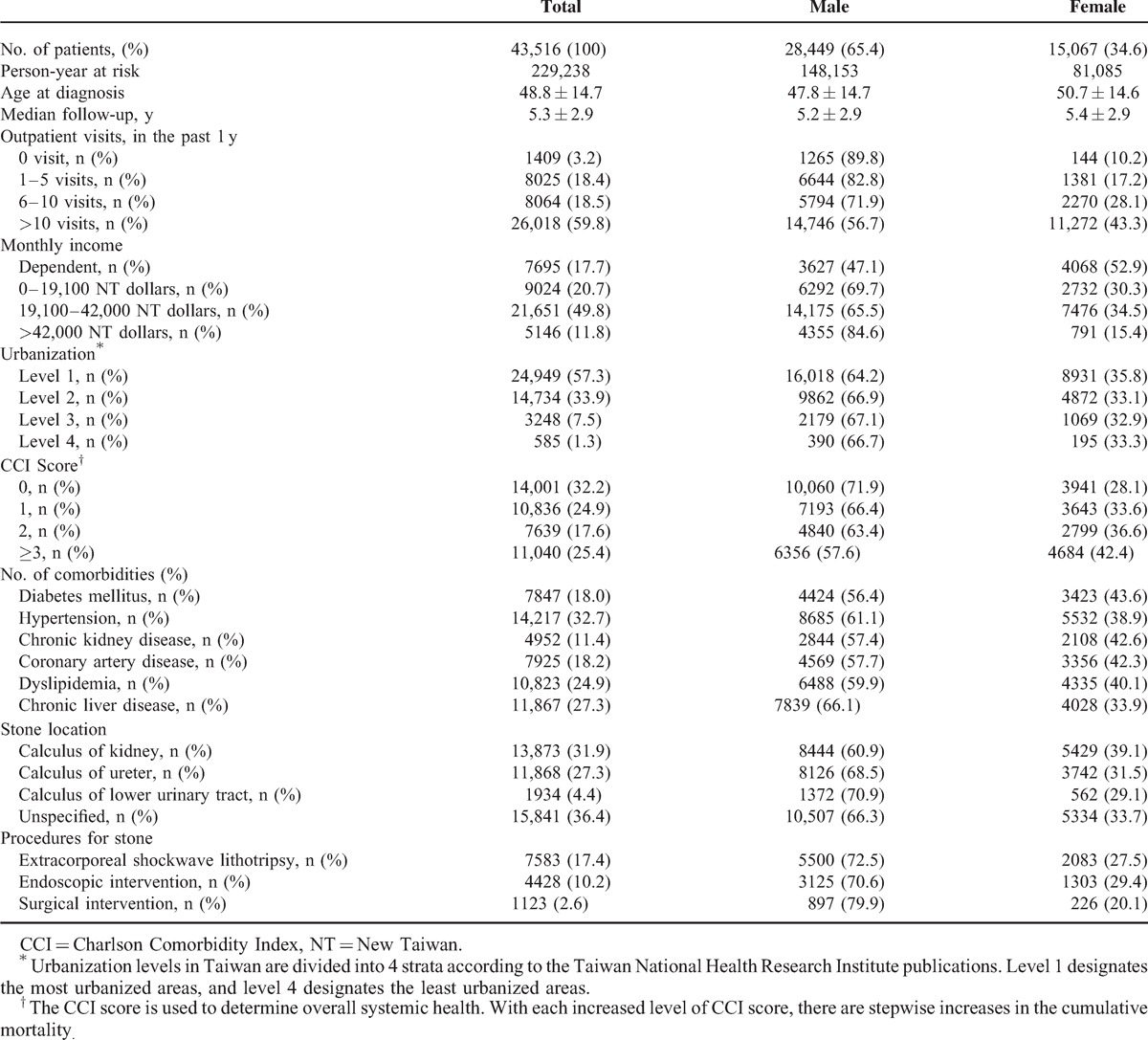
Characteristics of the Study Subjects

### SIRs of Cancer

During the follow-up period, 1891 cancers have developed. Compared with the general population, patients with urinary calculi were significantly associated with a higher risk of overall cancer (SIR 1.75; 95% CI, 1.68–1.83; *P* < 0.001). The risk of all cancers was found to be significantly increased in both men and women, SIR 1.91 (95% CI, 1.80–2.01, *P* < 0.001) and SIR 1.49 (95% CI, 1.38–1.62, *P* < 0.001), respectively (Table 2).

**TABLE 2:**
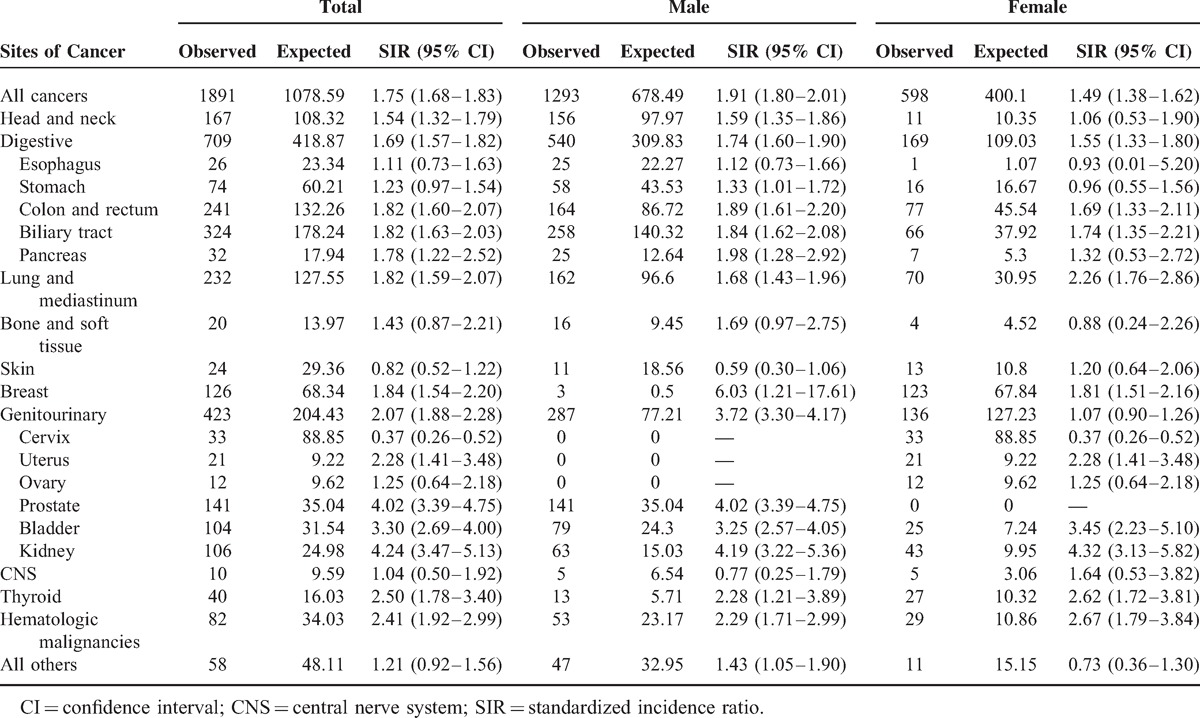
SIRs for Cancers in Patients with Urinary Calculi 1 More Year After Diagnosis

In subgroup analysis according to age, the SIRs were higher in patients aged 18 to 40 years (SIR 2.23; 95% CI, 1.88–2.62) than those in patients aged >40 years. When stratified by duration of urinary calculi, the SIRs were higher in patients with disease duration of 5 or more years. When stratified by stone location and treatment procedure, the SIRs were similar in all subgroups of patients (Table 3 and Supplementary Tables S1–S15, http://links.lww.com/MD/A121).

**TABLE 3:**
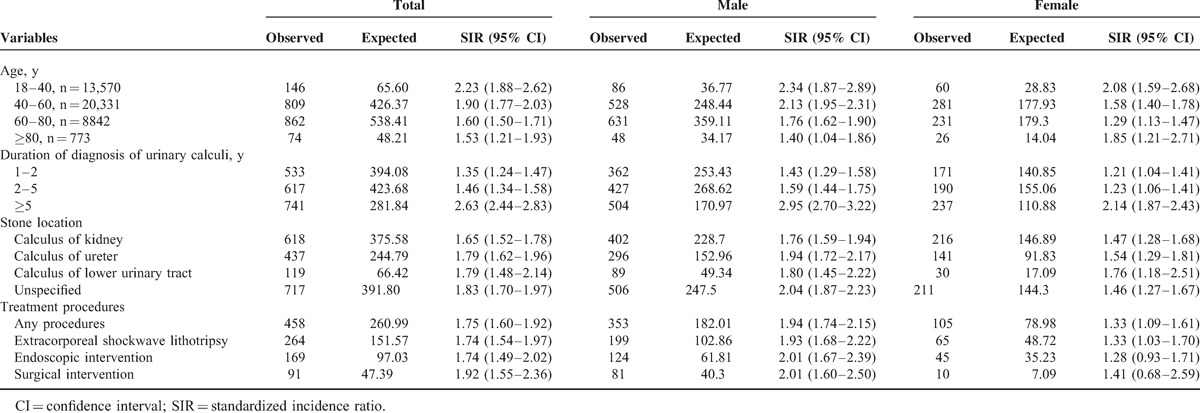
SIRs for Cancers Stratified by Age, Sex, Duration, Location, and Procedures of Urinary Calculi Disease

### Risk Factors for Cancer in Patients With Urinary Calculi

In multivariate analysis (Table 4), age (hazard ratio [HR], 1.05 for being 1 year older; 95% CI, 1.05–1.06; *P* < 0.001), male (HR, 1.36; 95% CI, 1.22–1.50; *P* < 0.001), CCI score (HR, 1.08 for 1 score increase; 95% CI, 1.05–1.11; *P* < 0.001), hypertension (HR, 1.12; 95% CI, 1.01–1.25; *P* = 0.040), chronic liver disease (HR, 1.43; 95% CI, 1.28–1.59; *P* < 0.001), low medical utilization (HR, 1.51; 95% CI, 1.14–1.99; *P* = 0.004), and low economic status (HR, 1.30; 95% CI, 1.07–1.59; *P* = 0.010) were found to be significant risk factors for cancer. The risk of cancer did not differ between stone locations in kidney, ureter, or lower urinary tract. The similar risk of cancer for patients with urinary calculi who receive extracorporeal shockwave lithotripsy, endoscopic, or surgical intervention was also noted. Compared with untreated patients, those who received treatment procedures for stone still had a similar risk of developing cancer (Supplementary Table S16, http://links.lww.com/MD/A121).

**TABLE 4:**
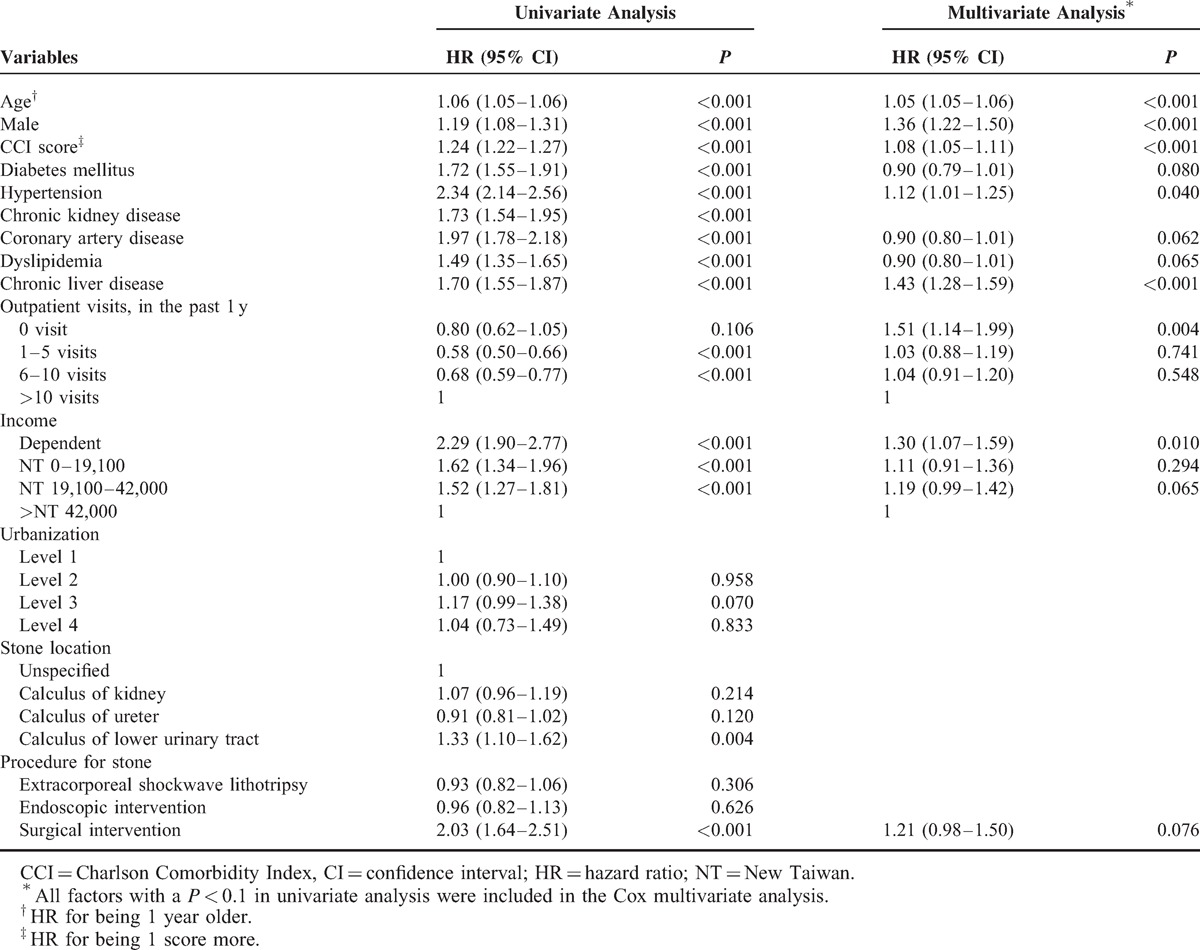
Risk Factors for Cancer in Patients with Urinary Calculi

### Sensitivity Analysis

We performed sensitivity analysis to examine the robustness of our results and confirmed the risk of cancers in patients with urinary calculi in different model parameters (Table 5). SIRs were consistently increased whether the study subjects were enrolled (only those who had no cancer occurrence within 90 days or 180 days). When the data were stratified according to treatment procedures for urinary calculi and different cohorts, we still found a consistent increase in the risk of cancers in patients with urinary calculi.

**TABLE 5:**
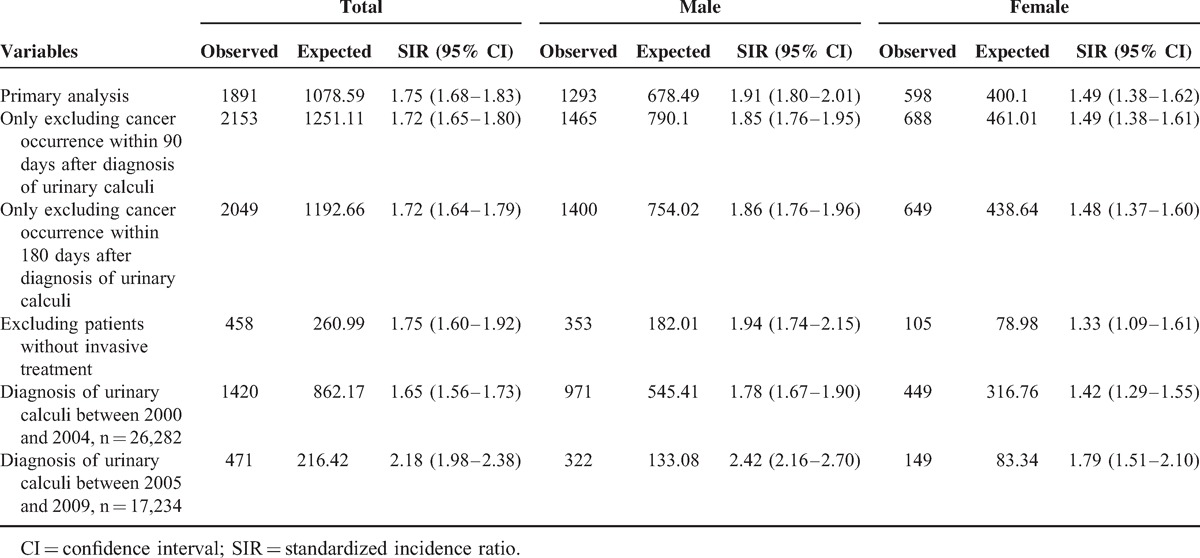
Sensitivity Analysis for Cancer Risk in Patients with Urinary Calculi Disease

## DISCUSSION

In the nationwide population-based study, our prevailing finding is the significantly increased risks of most common cancers in both male and female patients with urinary calculi compared with the general population in addition to the kidney and bladder cancers (Table 1). In subgroup analysis, the risk of cancers consistently increased regardless of stratification by age, duration, locations, or interventions of urinary calculi. In particular, low medical utilization and low economic status were found to be significantly correlated with cancer development in a multivariate analysis after adjusting for age, sex, and comorbidities.

Several studies regarding the association between urinary calculi and urinary tract cancers have been conducted, and the results showed that the increased risks of cancers ranged from 1.4 to 3.42 times as compared with the general population or matched controls.^8,9,24^ In 1997, Chow et al^8^ assessed the association of urinary tract cancers with kidney or ureteral stones in a population-based cohort study, in which only hospitalized patients were included. SIRs for kidney and bladder cancers were 2.5 and 1.4, respectively, and were lower than those in our study (SIR 4.2 and 3.3, respectively). This discrepancy may be due to only the inpatient data used in the study of Chow et al.^8^ Actually many patients with asymptomatic urinary calculi received outpatient clinic follow-up. To more accurately interpret this association, we enrolled both inpatient and outpatient data with at least twice the diagnosis of urinary calculi. Thus, our study may truly reflect the risk of cancers of kidney and bladder.

To date, to the best of our knowledge, no large-scale study was performed to analyze the impact of urinary calculi on overall cancer risk. In addition to an increase risk of urinary tract cancers similar to previous studies,^8,9,24^ our study also found that patients with urinary calculi significantly had a higher risk of cancers of thyroid (SIR 2.50), hematologic origin (SIR 2.41), breast (SIR 1.84), lung (SIR 1.82), digestive tract (SIR 1.69), and head and neck (SIR 1.54). A possible explanation for the association between urinary calculi and cancers is that the relatively high levels of chemicals or carcinogens induced by urinary calculi formation facilitate the tumor microenvironment interaction as shown in the animal studies.^6^ Another proposed mechanism is chronic inflammation induced by urinary calculi. Some mediators and cytokines represent a possible link between chronic inflammation and cancer development.^16,25^ In experimental studies, calcitonin gene related peptides released from obstructed kidney were associated with tumor angiogenesis and growth.^26,27^ In human studies,^28–30^ patients with urinary calculi had elevated acute phase reactants, such as tumor necrosis factor α and interleukin-1 and -6, which may induce tumor growth and metastasis in different types of cancers.^31,32^ Therefore, it is speculated that urinary calculi might cause local irritation to uroepithelium and further induce systemic tumorigenesis via concentrated carcinogens and inflammatory cascades. In subgroup analysis of our study, the longer the exposure duration of urinary calculi, the higher the SIR of cancer development. It is implicated to be associated with a higher burden of carcinogen and inflammation. However, further studies are still needed to clarify this finding.

We further found that patients aged 18 to 40 years had higher rate of cancer (SIR 2.23) than those aged >40 years did. In other words, as patients with urinary calculi got older, after 40 years age, the impact of urinary calculi on cancer incidence became less dominant, but it was still associated with a higher risk of developing cancer compared with the general population. One possible explanation for this is that the influence of urinary calculi on cancer in the elderly was not as substantial as the aging process and higher burden of comorbidities, which may increase cancer incidence.^33^ SIRs of cancer in different stone locations and treatment procedures for urinary calculi were also calculated, respectively. Intriguingly, the risk of overall cancer was consistently significant along with urinary tract irrespective of the location of urinary calculi. Moreover, even after treatment for urinary calculi, the risk of cancer was still higher than that in the general population. Possible explanation might be that urinary calculi could induce systemic inflammation instead of chronic irritation alone, and, therefore, it was not helpful to reduce the risk of cancers even after removal of the local irritated source.^10,28^

Cancer and urinary calculi, so-called modern diseases, cause prominent public health burden and financial expenditure. According to the World Health Organization report in 2008, cancer is one of the leading causes of death worldwide, with an estimated 7.6 million deaths (around 13% of all death).^34^ Cancer is also the first leading cause of global economic loss up to 895 billion dollars, which is higher than heart disease (753 billion dollars) in 2008.^35^ Several researches tried hard to find out risk factors and underlying mechanisms of cancer development to take effective and early steps to prevent its occurrence. Our large-scale population-based study revealed the significant association between urinary calculi and systemic cancers in addition to urinary tract cancers. In other words, careful evaluation is necessary to detect urinary calculi earlier because of some patients with only mild or even no symptoms. We should pay more attentions to the patients with urinary calculi; otherwise, they will face higher than average risk of cancers, which will lead to more enormous cost either in health or economic way.

Some limitations of our study should be addressed. First, the diagnostic biases of both urinary calculi and cancer were derived from administrative claims data reported by physicians and hospitals. These data may not be as accurate as diagnoses made by standardized protocol. Nevertheless, urinary calculi validated by intervention procedures were still associated with an increased risk of cancers. For cancer verification, data relied on Cancer Catastrophic Illness Certificate for that pathologic evidence were necessary, and laboratory and imaging data must be peer reviewed. Second, surveillance bias may lead to errors in reporting possibly unrelated cancer simply due to a more frequent use of high-resolution imaging studies in patients with urinary calculi. To minimize potential bias, we excluded newly diagnosed cancer within the first year of follow-up and a significantly increased risk of cancers was still noted even after a 5-year follow-up. Third, several potential confounding factors including obesity, tobacco use, alcohol, environmental exposure, and family history of cancer were not available in our analyses. Thus, the study outcomes may likely be altered by lack of those uncollected information in a large claims database. Fourth, causal relationship could not be answered in the current study, and further controlled studies would be warranted to validate the association.

In conclusion, our study demonstrated that patients with urinary calculi had a greater risk of developing systemic cancers. Although cost-effectiveness of active surveillance for occult cancer in patients with urinary calculi has not been determined, our results may identify a potential population with a higher risk of cancer. Further studies are needed to clarify the causal relationship and carcinogenic mechanisms involved.
